# Motivational strategies reduce recidivism and enhance treatment adherence in intimate partner violence perpetrators with substance use problems

**DOI:** 10.3389/fpsyt.2025.1538050

**Published:** 2025-01-30

**Authors:** Marisol Lila, Cristina Expósito-Álvarez, Manuel Roldán-Pardo

**Affiliations:** Department of Social Psychology, Faculty of Psychology and Speech Therapy, University of Valencia, Valencia, Spain

**Keywords:** intimate partner violence perpetrators, intervention programs, motivational strategies, individualized motivational plan, substance use problems

## Abstract

**Introduction:**

The incorporation of motivational strategies has shown promising results in increasing the effectiveness of intervention programs for intimate partner violence perpetrators, such as enhancing treatment adherence and decreasing risk of intimate partner violence recidivism. This could be particularly important for participants with alcohol and/or other drug use problems (ADUPs), who are at higher risk of recidivating and dropping out from the intervention. Consequently, there is a need to study whether motivational strategies are also effective for high-risk and highly resistant participants. The aim of this study was to determine whether the incorporation of motivational strategies led to improved outcomes in participants with ADUPs compared to those without.

**Methods:**

Participants were intimate partner violence male perpetrators who received a standard intervention (*n* = 349) or a standard intervention adding an individualized motivational plan (*n* = 367). Data on official intimate partner violence recidivism, intervention dose, and dropout were collected after the end of the intervention. Comparisons were made between participants with and without ADUPs in each intervention condition.

**Results:**

Results showed that in the full sample of participants, irrespective of their condition, those with ADUPs presented a higher recidivism (*p* = .007) and dropout rate (*p* = .003) and lower intervention dose than those without ADUPs (*p* = .005). When only considering participants in the standard intervention, results also showed that intimate partner violence perpetrators with ADUPs had a higher recidivism (*p* = .025) and dropout rate (*p* = .015) and lower intervention dose (*p* = .048) than those without. However, there were no significant differences between participants with and without ADUPs in the standard intervention adding an individualized motivational plan.

**Discussion:**

When incorporating motivational strategies into the standard interventions for intimate partner violence perpetrators, disparities between participants with and without ADUPs were mitigated. Specifically, participants with ADUPs showed similar outcomes to those without ADUPs after receiving the standard intervention adding an individualized motivational plan. Our results suggest that motivational strategies may be effective in reducing intimate partner violence recidivism and improving treatment adherence in high-risk and highly resistant intimate partner violence perpetrators.

## Introduction

Intimate partner violence (IPV) represents the most prevalent form of violence against women worldwide, with severe consequences for women’s health and wellbeing, and for the welfare of children and the broader societal structure (1). IPV has been recognized as a human rights and social problem of pandemic proportions (2, 3). Specifically, 27% of ever-partnered women aged 15-29 have suffered physical and/or sexual violence from their current or former male intimate partner at least once in their lifetime (2).

Given that IPV perpetrators often exhibit patterns of repeated abuse, either involving multiple victims or maintaining relationships with the same victim, it remains crucial to prevent IPV through direct intervention with perpetrators (4, 5). Intervention programs for IPV perpetrators have been developed in response to the recognition of IPV as a pervasive social issue, aiming to facilitate change in men and reduce IPV recidivism (6, 7). These programs represent a fundamental strategy within the criminal justice system to mitigate IPV among men convicted of such offenses, often providing an alternative to incarceration by court-mandating individuals to attend community-based intervention programs for IPV perpetrators (8, 9). As a result, there has been a growing interest in evaluating the effectiveness of perpetrator programs (10–12). However, significant challenges remain, with limited evidence supporting the effectiveness of these interventions, as highlighted in systematic reviews and meta-analyses (13–16).

The group-based structure of intervention programs for IPV perpetrators has often relied on a “one-size-fits-all” model, in which standardized interventions are applied uniformly without addressing the specific risk factors associated with IPV. In response, well-established approaches, such as the Risk-Needs-Responsivity (RNR) approach (17) and the Principles of Effective Interventions [(PEI) (18)], underscore the importance of conducting rigorous risk assessments and developing individualized strategies that address the unique risks and needs of each participant (15). These approaches offer a more sensitive and responsive intervention framework, aiming to improve outcomes by aligning program strategies with the participants’ specific needs (19, 20). Specifically, the RNR model, traditionally applied to criminogenic risk reduction, targets the “Big Eight” criminogenic risks domains, including history of antisocial behavior, antisocial personality and associates, family/marital relationship problems, substance abuse, employment, education and leisure problems (21). The core principles of the RNR framework can be articulated as follows: the ‘risk’ principle addresses *who* should be prioritized for treatment, the ‘need’ principle focuses on *what* specific factors should be targeted during intervention, and the ‘responsivity’ principle emphasizes *how* treatment should be delivered to maximize effectiveness (17). While the RNR framework is well-established, its application specifically to IPV remains more nascent. As such, it remains crucial to carefully tailor intervention programs for IPV perpetrators to the assessed needs and risk levels of participants (22). Research indicates that the RNR and PEI approaches show promise in reducing IPV recidivism more effectively than standardized models (15, 23–25).

Another critical challenge for perpetrator programs is effectively engaging high-risk IPV perpetrators who exhibit significant resistance to treatment (26–28). Notably, IPV perpetrators with alcohol and/or other drug use problems (ADUPs) constitute a high-risk, highly resistant group of IPV perpetrators (29, 30). This group not only demonstrates lower levels of treatment engagement but also tends to perpetrate more severe violence and has higher rates of dropout and recidivism than those without ADUPs (31). This is of particular concern given that approximately 50% of participants in perpetrator programs have ADUPs (32, 33). In addition, this group of participants present with complex, multi-level risk factors for IPV associated with ADUPs. At the individual level, participants with ADUPs often present lower anger management skills, diminished cognitive abilities, and increased psychological distress (34–36). Indeed, and consistent with the self-medication hypothesis (37), participants may use alcohol or other drugs to alleviate emotional pain (38, 39). In turn, ADUPs may exacerbate mental health issues through their impact on emotional and cognitive processes and on self-regulation (40). Moreover, at the social-relational level, participants with ADUPs are more likely to have experienced childhood trauma, complex relational trauma, and limited intimate support (34, 41–44). In addition, at the attitudinal level, participants with ADUPs frequently attribute responsibility for their violent behaviors to their substance use (45). Consequently, participants with ADUPs may exhibit greater individual, social-relational and attitudinal specific risk factors that warrant attention within intervention programs (30, 35). Improving outcomes for these participants, particularly by increasing their treatment engagement and reducing their elevated risk of IPV recidivism, could significantly enhance the overall effectiveness of perpetrator programs. Achieving this, however, requires rigorous evaluation of specific intervention strategies that show promise for this high-risk group of perpetrators (19, 23).

One of the key intervention strategies gaining attention is the incorporation of motivational-based approaches, which have been identified as a promising way to overcome intervention challenges and enhance the effectiveness of intervention programs for IPV perpetrators (46–48). For example, a recent systematic review by Wilson et al. (16) found inconclusive results regarding the overall effectiveness of IPV programs but highlighted the encouraging potential of integrating motivational strategies in newer programs. Originally developed in the field of addiction, motivational strategies have proven effective with highly resistant patients in addiction treatment (49). These strategies adopt a person-centered, collaborative approach designed to address ambivalence towards change (50). In addition, motivational strategies are grounded in four key principles: 1) expressing empathy and exploring the participant’s internal emotions, 2) evoking discrepancies between their values, goals, and current behaviors, 3) acknowledging and working with resistance rather than engaging in direct confrontation, and 4) enhancing the participant’s resources and strengths to bolster their confidence in their ability to change (51).

When incorporated into intervention programs for IPV perpetrators, motivational strategies involve specific humanistic techniques, including motivational interviewing (49), retention techniques (52, 53) and strength-based approaches such as the Good Lives Model (54), alongside the solution-focused brief therapy (55), goal setting (56–58), and the transtheoretical model of change (59). The transtheoretical model of change (59) outlines how individuals move through a series of stages when working toward intentional behavior change: precontemplation (resistance to change and avoidance of information), contemplation (ambivalence about change), preparation (taking concrete steps toward change), action (actively modifying behavior and implementing strategies for change), and maintenance (sustaining changes and preventing relapse). By aligning intervention strategies with the individual’s stage of change, these approaches may help promote greater treatment engagement and sustained progress throughout the intervention process (60).

Systematic reviews and meta-analysis assessing the effectiveness of motivational strategies demonstrate that integrating these approaches into perpetrator intervention programs can lead to improved treatment outcomes (14, 15). Indeed, a growing body of research suggests that incorporating motivational components enhances program effectiveness by fostering treatment engagement, strengthening participants’ motivation to change, and reducing dropout and recidivism rates (5, 61–65). For instance, recent meta-analyses have shown that participants receiving motivational strategies exhibited lower dropout rates than those attending standard perpetrators programs (48, 66).

Research suggests that the empathetic, non-confrontational nature of motivational strategies may facilitate that participants feel less resistant to the intervention, thus fostering treatment engagement (67). For instance, a study conducted by Crane and Eckhardt (61) showed that participants who received a brief motivational interviewing session prior to the intervention attended significantly more sessions than those in the control group. Furthermore, the Individualized Motivational Plan (IMP) developed by Lila et al. (68) has demonstrated greater effectiveness than standard interventions by increasing the intervention dose, facilitating progression in participants’ stages of change, and reducing physical IPV perpetration and risk of IPV recidivism among court-mandated male participants in perpetrator programs. Moreover, the use of individualized motivational strategies through the IMP could enable the intervention to be tailored to the specific needs of each participant (69).

Given that participants with ADUPs represent a high-risk and highly resistant group of perpetrators, this group of participants may particularly benefit from the IMP. This framework integrates both individual and group work within a humanistic, collaborative framework aimed at enhancing motivation to change and reducing resistance to intervention (67, 70, 71). However, while existing evidence suggests that the IMP can be effective for IPV perpetrators, further research is needed to assess the specific impact of these motivational strategies within this high-risk population (32, 72, 73). The aim of this study was to evaluate whether incorporating an IMP into a standard perpetrator program led to improved intervention outcomes, specifically, dropout rates, intervention dose, risk of IPV recidivism and official IPV recidivism, among IPV perpetrators with ADUPs. We hypothesized that the IMP would help reduce the disparities in these outcomes between IPV perpetrators with and without ADUPs.

## Method

### Participants

The sample consisted of 716 men who were court-mandated to participate in a community-based intervention program for IPV perpetrators at the University of Valencia [Contexto Program (68)]. Participants were part of 73 intervention groups conducted between November 2012 and October 2022. The sample included only those participants who met the following criteria: a) age 18 or older, b) convicted of IPV and court-referred to participate in an intervention program for IPV perpetrators, c) absence of serious mental health problems and/or disruptive behavior, and d) signed the informed consent form. Regarding the participants’ sociodemographic characteristics (see **Table 1** for a more detailed description), the mean age was 39.5 (*SD* = 11.3). Approximately one-third of the participants (28.6%) had immigrant status. Specifically, 14.7% were from Latin America, 7.1% were from Europe (except Spain), 5.4% were from Africa, and 1.4% were from Asia. Regarding the educational level, 7.4% had no studies, 45.5% had completed elementary studies, 36.9% had obtained a high school diploma, and 10.2% had completed a college degree. Most of the participants were not partnered: 35.6% were single, 12.3% were separated, 26.5% were divorced, and 0.60% were widowed. The remaining (25%) were married or in a partnership. The unemployment rate among participants reached 40.6%, while the median annual family household income fell between €6000 and €12000.

**Table 1:** Sample sociodemographic characteristics.

Variables	*M*(*SD*)	Range	*n*(%)
Age	39.5 (11.3)	18-81	
Annual income^1^	4.25 (2.41)	1-12	
Origin			
Spain			511 (71.4)
Latin America			105 (14.7)
Europe (except Spain)			51 (7.1)
Africa			39 (5.4)
Asia			10 (1.4)
Level of education			
No education			53 (7.40)
Elementary			326 (45.5)
High School			264 (36.9)
College			73 (10.2)
Marital status			
Married or with partner			179 (25.0)
Single			35.6 (35.6)
Separated			88 (12.3)
Divorced			190 (26.5)
Widowed			4 (0.60)
Employed			
Yes			425 (59.4)
No			291 (40.6)

*M*, Mean; *SD*, Standard Deviation.

^1^Annual income: 1 = <€1800, 2 = €1800-€3600, 3 = €3600-€6000, 4 = €6000-€12000, 5 = 12000-€18000, 6 = €18000-€24000, 7 = €24000-€30000, 8 = €30000-€36000, 9 = €36000-€60000, 10 = €60,000–€90,000, 11 = €90,000–€120,000, and 12 = >€120,000.

### Instruments

*Dropout.* A value of 0 was assigned to participants who completed the intervention program, while a value of 1 was assigned to those who ceased attendance.

*Intervention dose.* The ratio of the number of sessions attended by the participant to the total number of sessions in the intervention group.

*Official IPV recidivism.* Data on recidivism rates were obtained from VioGén (74), the monitoring system of the Ministry of Home Affairs. The system provides data on any new incident of IPV, formal complaint, or breach of the conditions prescribed by the judge (e.g., restraining order). The data is derived from all the institutions engaged in the provision of victim protection services. Data were collected in May 2024. Any new incident occurring within 12 months of the conclusion of the intervention was deemed to be recidivism.

*Risk of recidivism evaluated by the facilitators.* The Spousal Assault Risk Assessment Guide [SARA (75); Spanish version by Andrés-Pueyo et al. (76)] was employed to evaluate the risk of recidivism. The SARA is completed by the facilitators and comprises a 20-item checklist protocol on a 3-point Likert-type scale (0 = *absent*, 1 = *possibly present*, and 2 = *present*). SARA also comprises two independent items designed to assess the risk of recidivism towards a partner and others. These items are presented on a 3-point Likert-type scale (0 = *low risk*, 1 = *moderate risk*, and 2 = *high risk*). In the present study, only these two items were utilized. The SARA has been shown to have predictive validity (77), and the Spanish version has been employed in research with samples of IPV perpetrators (68, 78).

*Alcohol use.* The Alcohol Use Disorders Identification Test [AUDIT (79); Spanish version by Contel-Guillamón et al. (80)] was used to screen the level and frequency of current and past 12-month alcohol use. The instrument comprises 10 Likert-type items, which are scored on a 3-point or 5-point scale (for example, 0 = *never*, 1 = *less than once in a month*, 2 = *once a month*, 3 = *once a week*, and 4 = *daily or almost daily*). A score of 8 or above was indicative of hazardous alcohol use. The AUDIT has been shown to have construct and discriminant validity and sensitivity (81), and the Spanish version has been employed in studies of IPV male perpetrators (78, 82). In the present study, Cronbach’s alpha reliability coefficient was .78.

*Clinical syndromes of alcohol and substance dependence.* The alcohol dependence and substance dependence scales of the Millon Clinical Multiaxial Inventory-III [MCMI-III (83); Spanish version by Cardenal and Sánchez (84)] were used to screen participants for alcohol and/or other drug dependence. The self-reported inventory consists of 175 true-false items, and only the two aforementioned scales were used. A score of 75 or above was indicative of a potential issue or proclivity towards a clinical dependence syndrome. The Spanish version of the scale has been utilized in studies involving male perpetrators of IPV (28, 57). The Cronbach’s alpha reliability coefficients for alcohol dependence and substance dependence were .71 and .80, respectively (84).

### Procedure

Men convicted of IPV who consented to participate in the present study signed a written consent form during the assessment phase. It was explicitly conveyed to participants that neither their decision to participate nor their refusal to do so would have any bearing on their legal standing. Participants were assigned to a treatment condition based on the availability and training of the facilitators. While all facilitators were trained to deliver the standard intervention (SI) condition, a specific group of facilitators were also trained to deliver the standard intervention with the Individualized Motivational Plan (SI-IMP) condition. The SI condition comprised 70 hours of intervention (35 weekly two-hour group sessions) based on cognitive-behavioral techniques addressing topics such as sexism, gender equality, and IPV. The six-module program sought to cultivate group trust, address IPV responsibility, enhance communication, empathy, and emotion management, challenge traditional gender roles, and prevent relapses. The SI-IMP condition comprised the standard intervention in conjunction with the IMP. The IMP employed a package of motivational strategies aimed at enhancing treatment compliance and motivation to change. Based on approaches such as motivational interviewing and the Good Lives Model, it included five individual sessions, three group sessions for goal sharing, ongoing facilitator reinforcement, and retention techniques throughout the intervention. For a more detailed description of each of the treatment conditions, refer to Lila et al. (68). In total, 349 participants received the SI, and 367 received the SI-IMP.

Data on sociodemographic characteristics, alcohol use, and clinical syndromes of alcohol and substance dependence were collected as a part of the initial assessment for participants. This data was gathered through a self-report assessment instrument administered by the program staff over two sessions, each spanning two hours. After the end of the assessment phase, facilitators reported data on the risk of IPV recidivism. Individuals who scored above the established cut-off point on the AUDIT [≥ 8 (79)] or the alcohol or substance dependence scale of the MCMI-III [≥ 75 (83)] at the time of intake were classified as ADUPs. This procedure has been utilized previously in IPV male perpetrators studies (34, 78). Profile validity of the MCMI-III was evaluated using its validity scale. When invalid profiles were detected, the assessment was readministered to ensure accurate and reliable results. Finally, data on dropout, official recidivism, and intervention dose were collected subsequent to the conclusion of the intervention period. Dropout and intervention dose data were reported by the facilitators, while the research staff were responsible for collecting the data on official recidivism. The data presented in this study were collected in accordance with the approved procedures established by the Ethics Committee of the University of Valencia (H1537520365110).

### Data analysis

First, the baseline characteristics of the participants in each treatment condition (SI vs. SI-IMP) were examined. Sociodemographic characteristics and risk of IPV recidivism were included. Chi-square and *t* tests were used to compare categorical and continuous variables, respectively, across treatment conditions. Continuous variables with non-normal distributions were analyzed using the Mann-Whitney U test.

Second, different analyses were conducted to analyze the differences between participants with and without ADUPs. Chi-square and Mann-Whitney U tests were conducted for categorical (i.e., dropout and official IPV recidivism) and continuous (i.e., intervention dose) variables, respectively. The effect size for categorical and continuous variables was calculated using Cramér’s *V* and eta-squared statistics, respectively. These analyses were performed for the full sample and each of the treatment conditions. All analyses were conducted using the IBM SPSS statistical software, version 28.0.1.1.

## Results

### Baseline characteristics

**Table 2** provides a comparison of the baseline characteristics and risk of IPV assessed by facilitators of the participants who were allocated to each treatment condition. Seven variables were compared between groups, and none of the comparisons reached statistical significance (*p* >.05). Therefore, the groups were found to be statistically equivalent in terms of their baseline characteristics and risk of IPV.

**Table 2:** Comparison of baseline characteristics and risk of IPV.

Variables	SI (*n* = 349)			SI-IMP (*n* = 367)			
	*M*	*SD*	*%*	*M*	*SD*	*%*	*t / Z / χ^2^*
Risk of IPV	0.55	0.67		0.65	0.78		-1.33
Risk of general violence	0.30	0.51		0.35	0.64		-0.24
Age	39.1	11.6		39.9	11.1		-1.55
Immigrant status							1.79
Yes			30.9			26.4	
No			69.1			73.6	
Educational level							3.65
No education			8.90			6.00	
Primary			46.7			44.4	
Secondary			34.1			39.5	
University			10.3			10.1	
Current partner							0.67
Yes			26.4			23.7	
No			73.6			76.3	
Employment							2.72
Yes			62.5			56.4	
No			37.5			43.6	

IPV, Intimate Partner Violence; SI, Standard Intervention; SI-IMP, Standard Intervention – Individualized Motivational Plan; *M*, Mean; *SD*, Standard Deviation; *t*, independent *t* test; *Z*, Mann-Whitney *U* test; *χ^2^*, chi-square test.

All comparisons were non-significant at the .05 level.

### Final outcomes

**Table 3** presents the descriptive statistics of the final outcomes for the full sample and for each treatment condition. In consideration of the full sample, participants with ADUPs exhibited elevated rates of dropout (*χ^2^* = 8.58; *p* = .003; *V* = .11) and IPV recidivism (*χ^2^* = 7.18; *p* = .007; *V* = .10), as well as a diminished intervention dose (*Z* = -2.78; *p* = .005; *d* = .38), in comparison to the participants without ADUPs. When only the participants assigned to the SI condition were considered, it was observed that those with ADUPs also demonstrated elevated rates of dropout (*χ^2^* = 5.87; *p* = .015; *V* = .13) and recidivism (*χ^2^* = 4.99; *p* = .025; *V* = .12), as well as a diminished intervention dose (*Z* = -1.97; *p* = .048; *d* = .47), in comparison to those without ADUPs. However, when considering only participants assigned to the SI-IMP condition, no statistically significant differences were observed in dropout, IPV recidivism, and intervention dose (*p* >.05).

**Table 3:** Descriptive statistics on final outcomes in the full sample and each treatment condition.

Variables	Full sample				SI condition				SI-IMP condition			
	ADUPs (*n* = 237)		W-ADUPs (*n* = 479)		ADUPs (*n* = 118)		W-ADUPs (*n* = 231)		ADUPs (*n* = 119)		W-ADUPs (*n* = 248)	
	*n/M*	%/*SD*	*n/M*	%/*SD*	*n/M*	%/*SD*	*n/M*	%/*SD*	*n/M*	%/*SD*	*n/M*	%/*SD*
Dropout												
Yes	55	23.2	69	14.4	26	22.0	28	12.1	29	24.4	41	16.5
No	182	76.6	410	85.6	92	78.0	203	87.9	90	75.6	207	83.5
Recidivism												
Yes	28	11.8	29	6.1	10	8.5	7	3.0	18	15.1	22	8.9
No	209	88.2	450	93.9	108	91.5	224	97.0	101	84.9	226	91.1
Intervention dose	.76	.29	.84	.42	.75	.27	.84	.54	.77	.32	.83	.27

SI, Standard Intervention; SI-IMP, Standard Intervention – Individualized Motivational Plan; ADUPs, Alcohol and/or Drug Use Problems; W-ADUPs, Without - Alcohol and/or Drug Use Problems; *n*, number; *M*, Mean; *SD*, Standard Deviation.

## Discussion

This study aimed to evaluate whether the incorporation of a motivational tool, the IMP, helped reduce risk factors between IPV perpetrators with ADUPs and those without, in a large sample of men court-mandated to attend an intervention program for IPV perpetrators. Our results indicated that, both in the full sample and specifically within the standard condition (SI), participants with ADUPs showed higher dropout rates, increased official IPV recidivism, and received a lower intervention dose (i.e., lower treatment attendance) compared to participants without ADUPs. This is consistent with previous literature showing that presenting ADUPs among IPV perpetrators was predictive of higher dropout and lower treatment adherence (29, 85–87). In this vein, a study conducted by Expósito-Álvarez et al. (57) revealed that perpetrator programs’ participants with ADUPs had 123% higher odds of dropping out than those without such problems. This result is of significant concern given the well-known association between dropout and a higher likelihood of IPV recidivism (27, 29, 88). Moreover, and consistent with our results, IPV perpetrators with ADUPs have been consistently found to display higher IPV recidivism rates compared to those without ADUPs (31, 89). Nonetheless, it is important to note that while ADUPs may contribute to and exacerbate violence among IPV perpetrators, IPV is a complex phenomenon influenced by a range of multifaceted risk factors (90). These factors, which may interact with ADUPs, include emotional dysregulation, trauma, gendered power dynamics, and diminished executive functions, among others, all of which increase the likelihood of IPV (35, 41, 45, 91). Therefore, our findings, in alignment with existing literature, indicate that IPV perpetrators with ADUPs are a high-risk group of participants who may present specific risks that need to be targeted in perpetrator programs (92).

Importantly, our results also showed that when incorporating the IMP into the standard intervention (SI-IMP), participants with and without ADUPs did not differ in terms of intervention dose, dropout and official recidivism rates. This suggests that the disparities commonly observed between IPV perpetrators with and without ADUPs were mitigated when the IMP was integrated into the standard perpetrator program. The observed reduction in risk factors often associated with ADUPs may be attributed to the motivational strategies of the IMP, which are well-documented for their ability to enhance treatment engagement and improve retention in interventions (60, 63–65). Moreover, participants with ADUPs, who have been shown to exhibit higher levels of anger and impulsivity (34, 36, 93), may have particularly benefited from the motivational components of goal setting and strategies to help them identify personal reasons for change, thereby promoting more effective engagement in the intervention process and reducing the likelihood of IPV recidivism (56, 57, 94–96). Therefore, our findings underscore the encouraging potential of incorporating motivational strategies in perpetrator programs, particularly for high-risk participants such as those with ADUPs. Prior randomized controlled trials (RCTs) have also shown that implementing brief motivational interviewing sessions at intake may show promising results for IPV perpetrators with ADUPs compared to standard interventions (70, 71, 73).

In alignment with emerging frameworks, such as the RNR model (17) and the PEI approach (25), there is an urgent need for interventions that address the specific risks and needs of IPV perpetrators to effectively mitigate key risk factors, including ADUPs (18, 23). However, IPV perpetrators with co-occurring ADUPs have traditionally been referred to separate programs for IPV intervention and addiction treatment, often located in different facilities and administered by distinct services, which is both time- and cost-consuming (92, 97, 98). This lack of integrated approaches contributes to negative outcomes, including the high dropout rates and low treatment engagement often found in these participants (27, 29, 99). Integrated approaches that concurrently address IPV and ADUPs while incorporating motivational strategies, may offer a more effective approach to improve treatment outcomes among IPV perpetrators with ADUPs. In this vein, a recently published RCT has shown that incorporating an IMP tailored to addressing ADUPs was more effective than a standard IMP in reducing alcohol use, increasing active participation, and promoting more advanced stages of change among IPV perpetrators (78). These findings suggest that integrated, motivation-based interventions hold significant promise for addressing the needs of IPV perpetrators with co-occurring ADUPs (71–73). However, as Eckhardt et al. (97) recently emphasized: “*effective interventions that break this robust and complicated association are under development but are limited in number and availability*” (p. 2412). Thus, more intervention and research efforts are needed to implement motivational-based and integrated approaches in perpetrator programs.

Our findings have important treatment implications, since they revealed that IPV perpetrators with ADUPs exhibit a higher risk of official IPV recidivism, dropout, and lower treatment adherence, thus showing that this high-risk group of perpetrators require tailored interventions within group-based intervention programs (30, 31). In addition, integrating motivational strategies appears particularly beneficial in reducing their risk factors for IPV (71, 78). The person-centered, non-confrontational approach of motivational strategies may help these individuals resolve ambivalence toward change, given the potential discrepancy between their behavior (e.g., consequences of substance use, conflictive and violent behavior) and their core values (52, 54). In fact, previous studies indicate that IPV perpetrators with ADUPs demonstrate greater motivation and are often at a more advanced stage of change than those without ADUPs (34, 100). Thus, enhancing treatment engagement and promoting their internal motivation to change could foster positive change in their intimate relationship dynamics, ultimately decreasing their elevated risk of IPV recidivism (45, 47).

This study has certain limitations. Bivariate comparisons may not fully capture the complex relationships between variables and may overlook confounding factors that could impact outcomes across intervention conditions (101). Future studies should consider using RCTs to directly compare the effectiveness of these interventions in reducing risks associated with ADUPs in IPV perpetrators (68, 70, 78). Additionally, equivalence testing should be considered as a rigorous method to evaluate whether treatment outcomes are truly equivalent across groups (102). Future studies could also include a wider range of variables that reflect other risk factors for IPV associated with ADUPs, such as attitudinal and social-relational variables, beyond recidivism and treatment engagement (68, 78). Further research is also needed to assess the specific types of substances used by IPV perpetrators and their levels of use (e.g., hazardous drinking, alcohol dependence) to inform the design of more tailored intervention strategies for IPV perpetrators with ADUPs (103). Another limitation is that this study used a specific sample of men court-mandated to attend an intervention program for IPV perpetrators following a conviction for a gender-based violence offense, thus limiting the generalizability of these results to other populations. Additional research should focus on understudied problems such as IPV among men who have sex with men (5). This study also exhibited several strengths. Notably, it utilized a large sample size (*n* = 716), which is particularly significant given the challenges associated with recruiting participants from this population. In addition, while research on motivational approaches within perpetrator programs often focuses on the incorporation of motivational interviewing solely at intake, thereby limiting its sustained use across the intervention, this study evaluated the implementation of the IMP, which was integrated throughout the intervention. Specifically, the IMP used both individualized strategies and group-based methods for sharing goals and progress, potentially enhancing treatment engagement (65, 103).

In conclusion, IPV perpetrators with ADUPs represent a high-risk and highly resistant group of IPV perpetrators who require tailored intervention approaches. Integrating motivational strategies, such as the IMP, could enhance treatment outcomes by improving engagement and reducing IPV recidivism. Such improvements have the potential to increase the effectiveness of perpetrator programs, thereby contributing to greater safety for victims.

## Data Availability

The raw data supporting the conclusions of this article will be made available by the authors, without undue reservation.
